# Correction of Visual Perception Based on Neuro-Fuzzy Learning for the Humanoid Robot TEO

**DOI:** 10.3390/s18040972

**Published:** 2018-03-25

**Authors:** Juan Hernandez-Vicen, Santiago Martinez, Juan Miguel Garcia-Haro, Carlos Balaguer

**Affiliations:** Systems Engineering and Automation Department, Universidad Carlos III de Madrid, Avd. Universidad, 30, Leganés, 28903 Madrid, Spain; juanhernandezvicen@gmail.com (J.H.-V.); jgarciah@ing.uc3m.es (J.M.G.-H.); balaguer@ing.uc3m.es (C.B.)

**Keywords:** humanoid robot, artificial vision, non-grasping manipulation, Neuro-Fuzzy filter, distortion correction

## Abstract

New applications related to robotic manipulation or transportation tasks, with or without physical grasping, are continuously being developed. To perform these activities, the robot takes advantage of different kinds of perceptions. One of the key perceptions in robotics is vision. However, some problems related to image processing makes the application of visual information within robot control algorithms difficult. Camera-based systems have inherent errors that affect the quality and reliability of the information obtained. The need of correcting image distortion slows down image parameter computing, which decreases performance of control algorithms. In this paper, a new approach to correcting several sources of visual distortions on images in only one computing step is proposed. The goal of this system/algorithm is the computation of the tilt angle of an object transported by a robot, minimizing image inherent errors and increasing computing speed. After capturing the image, the computer system extracts the angle using a Fuzzy filter that corrects at the same time all possible distortions, obtaining the real angle in only one processing step. This filter has been developed by the means of Neuro-Fuzzy learning techniques, using datasets with information obtained from real experiments. In this way, the computing time has been decreased and the performance of the application has been improved. The resulting algorithm has been tried out experimentally in robot transportation tasks in the humanoid robot TEO (Task Environment Operator) from the University Carlos III of Madrid.

## 1. Introduction

Advances in robotics technology are encouraging the development of novel applications and new tasks to be accomplished by humanoid robots. Among these new tasks, we find those centered around manipulation, which can be classified into two main groups: grasping tasks, in which the way to grab an object by the hand of a humanoid robot is studied [1,2] and non-grasping tasks. In the latter, the object might be moved by another [3] or might be placed over an object that the robot is grasping. This second case is studied in this paper, in which it is considered that the manipulated object is not linked to the robot through a rigid union or joint.

The application developed to study this second case in the paper and perform the experiments was done with a bottle on a tray, as shown in Figure 1. Consequently, because there is a lack of solid union, it is more difficult to ensure a proper transportation task while preventing the object from falling. There are many useful features that can be applied in object balance control, such as its inclination angle or its angular rotation rate. The appropriate selection of features among all of them depends on the control methods and algorithms applied later on. Considering the object transported acting as a simple linear inverted pendulum over the tray, the most important piece of information will be the one related with its rotation. In this work, the rotation information, that is, the angle of the object is obtained from the visual system of the robot. However, this information cannot be used directly. It contains inaccuracies and errors found in every artificial vision system that would reduce the performance of the object balance controller. Therefore, it is important to obtain these features with a high level of accuracy in order to be capable of achieving a stable control of the bottle.

To obtain the object features, computer vision techniques have been applied. From the images obtained with the camera, the visual information has been transformed into the proper data needed to control the object. Unfortunately, this visual information is distorted by the inherent error of the camera and by the external errors introduced in the images (e.g., perspective). In the developed method, this has been applied to the example described before (the bottle on a tray), and those errors have been removed from the final information given to the robot in one fast single step. This task has been achieved thanks to the development of a Fuzzy filter with experimental data that enable the processing and combination of multiple parallel inputs at the same time. The correction of these errors has allowed us to achieve an improved control algorithm to maintain the stability of the transported bottle implemented in the robot TEO [4].

Before beginning the correction of the errors, it is recommended to highlight the similitude and differences between a human eye and a camera. Even though there are several similarities between both systems, some important differences can be observed. For example, the human eye is only capable of seeing objects defined in the foveal zone (the center of the vision field), and, in the peripheral field, everything is blurred Figure 2a, and Figure 2c. In contrast, the camera is capable of obtaining clear information from the complete image field Figure 2b. However, although the picture continues to be sharp in the peripheral field, a distortion from the camera can be detected. This distortion also appears in the human eye, but our brain is capable of correcting it, automatically distinguishing between an object that is straight and one with an inclination angle. However, the computer vision systems do not apply this correction automatically. Consequently, this distortion changes the values of the bottle features (inclination angle, etc.), making them unreal.

In this paper, the combination of three aspects will allow developing in the future an architecture based on a predictive control where the robot visual perception is the main base. To obtain such a predictive controller (Figure 3), a mathematical model is used to model the stability bottle behavior, that is, the first aspect. Therefore, the bottle has been modeled by considering it as a Linear Inverted Pendulum Model (LIPM) [5]. Through the Zero Moment Point (ZMP) balance criterion [6] and the LIPM model, the value of bottle instability $ZMP_{b.m.}$ will be calculated. The second one is the pose information of the bottle. The bottle pose is the estimated position and orientation ($\hat{\theta },{\hat{X}}_{CoG}$) of the Center of Geometry (CoG) of the bottle. This data is obtained from computer vision algorithms and processed by a Fuzzy filter to eliminate visual errors. The last one is the tray pose. This pose is the position and orientation of the Tool Center Point (TCP) of the arm limb ($P_{TCP}$). Through the robot’s kinematics, the pose (in the Cartesian space) of the support point of the bottle on the tray will be obtained.

Therefore, merging the bottle model with the stability information obtained from the Fuzzy filter and the tray pose of the arm will allow achieving a good control. These three aspects and the $ZMP_{ref\_bottle}$ are the inputs of the predictive controller, as it is shown in Figure 3. The output generated by the predictive controller is an estimated pose of the tray to correct the stability (${\hat{P}}_{TCP}$). This pose will be transformed to the joint space and applied on the joints of the robot (${\hat{q}}_{robot}$). The new joint positions will be returned to close the control loop.

This combination will enable the future development of an architecture based on a predictive control where the model of the bottle is the main base. To achieve such a predictive control, it is necessary to use a model in order to predict the bottle behaviour. Therefore, the bottle has been modelled by considering it as an inverted pendulum. Combining this with the information obtained by computer vision will allow us to achieve a good level of control.

Thus, the accuracy of the object features obtained in this research is important. The information related to the bottle equilibrium will be associated with the robot body pose and with the movement generated by disturbances on the robot. When a disturbance is applied to the humanoid robot, the whole robot behaves like a rigid solid and therefore the amount of movement produced by the disturbance is transmitted to the bottle. Thus, when the camera measures the bottle characteristics, the control system takes into consideration the perturbations on the robot. Finally, with all this information, the control system predicts the point at which the arm with the tray should reach out to stabilize the bottle.

At the University Carlos III of Madrid, the humanoid robotics group RoboticsLab has developed a new method to eliminate the distortion errors related to computer vision. This method allows the extraction of real movement values online and without having to rectify the captured images, which reduces the computation time.

The paper is organized as follows: the following section presents the problem to be solved by the experimental procedure discussed in the paper. In the third section, other existing techniques to solve the problem are presented, as well as the basics of the inverted pendulum and Neuro-Fuzzy learning, which is used to solve the error problems related to the information obtained by computer vision. In the fourth section, the methodology applied in the experiments is used to develop the proposed Fuzzy filter. In the fifth section, the experiments and results that demonstrate the correction of the error in the system proposed are shown. Finally, some conclusions and prospects for the future are shown.

## 2. Problem Statement

Humanoid robots whole body control is a multi-variable task that should be performed within short time cycles. Multiple sensor inputs are fed to the control algorithms that need the information in a proper way. Therefore, the data acquired by sensors must be filtered, adapted and combined to be useful for the controllers. These questions lead to the proposal of two problems to be studied related to the sensor information processing method. The first one is the need of performing a very fast data treatment to obtain the input data for the controller from the raw data from the sensors. The second problem is the existence of errors added to the sensor data caused by the hardware system.

Usually, humanoid controllers are based on simplifications of body structure and its dynamics to reduce computation time. This is the case of balance controllers that rely on simplifications such as the LIPM or the Cart-Table model [7] to optimize the control time. Therefore, considering the stability control of the transported object as the task goal, the behavior of the bottle on the tray can be considered similar to the behavior of an inverted pendulum. However, the use of any simplified model is supported by the use of the proper robot’s state information.

In the case discussed in this paper, the parameters of the model are obtained from robot vision sensors. The model input information (inclination angle, location of the object, etc.) is obtained by artificial vision techniques. The visual information processing requires a high level of computing effort and sequentially the application of different algorithms to obtain the proper data to be applied for the control. This *classic* way of visual data treatment is quite slow and constitutes an obstacle to improve balance controllers’ performance. The work described in this paper deals with this question by means of the development of a Fuzzy filter to process the visual information. As the first main achievement, it aims at reducing computing steps.

The other important point to consider is the inherent errors enclosed in the images obtained by computer vision. They have to be corrected if real information obtained from a camera is used as these inputs. When describing the errors introduced in systems based on computer vision, a classification of two groups can be made: errors produced by the camera defects, which are directly related to the camera lenses and errors caused by the perspective in which the object is being observed.

The camera defects are related to the nature of the lenses that are being used. Due to this fact, different kinds of radial deformations [8] in the image can be found, as seen in Figure 4. On one hand, if the focal length of the camera is short, the barrel deformation (a) will appear. In barrel distortion, image magnification decreases with distance from the optical axis. The apparent effect is that of an image which has been mapped around a sphere (or barrel). Whereas, on the other hand, if the focal length of the camera is long, a different deformation, named pincushion distortion (b), will appear. The image magnification increases with the distance from the optical axis. The visible effect is that lines that do not go through the centre of the image are bowed inwards, towards the centre of the image, like a cushion.

As the defects introduced by the camera lenses in our system are so low, they have been disregarded. However, high errors are introduced by the perspective in which the bottle is being observed by the camera at each moment. To understand this error, in Figure 5, two images can be seen. In both of them, the tray and the bottle are maintained in the same pose. The only variation has been made in the orientation of the TEO’s head. As it can be observed, the image changes completely, considering the bottle to be fully straight in one image (a) and with an inclination angle in the other (b). Even though in both cases the relative position between tray and bottle is the same, if the real tilt angle is aimed to be obtained directly from these images without using any filter, the data acquired in the first one would be similar to the real angle, whereas the second one would be interpreted wrongly, obtaining an erroneous inclination angle.

Thus, the Fuzzy filter developed and described in this work deals with the errors of the system at the same time that it reduces the computing time. This second feature of the Fuzzy filter is the second main achievement exposed in this work.

## 3. Background

In this section, we present various existing ways in which the error inherent from images can be corrected, how the bottle has been modeled to acquire the necessary characteristics, and bases of the tools used in this work to achieve the desired results.

### 3.1. Vision

A wide range of procedures is already available to correct the perspective error. For example, some of the techniques that we found implement a correction by comparing images obtained from different points of view (stereo-images) [9], whereas, in others, the correction is achieved by adjusting images from uncalibrated cameras [10]. Another example is the research developed in [11], in which the perspective error in different kinds of images is well corrected. However, in the above cases, the original image must be modified. These techniques were applied as a base for our first approach to obtain the real angle inclination of the bottle in the waiter application. However, different problems were found and the image rectification approach was discarded.

As mentioned previously, in the end, the real inclination angle of the bottle is needed to control its equilibrium. By using image rectification methods, the geometric image characteristics are modified. As a consequence, from this image, it is not possible to obtain any real information about the inclination angles without introducing errors, as shown in Figure 6 (top). Despite this, if the angle measurement error could be corrected, it would imply the use of other information provided by sensors other than the camera, such as kinematic estimations of the handling posture. The other inconvenience is the time complexity of the algorithm. In many research papers [12,13,14], different Hough transformations and computer vision algorithms are presented. Depending the algorithm and the amount of data processed, the minimum time complexity that can be achieved with the Hough transformation is $O{\left(N\right)}^{3}$. The pseudo-code that represents the basic procedure for angle calculation based image transformation techniques is exposed in the Algorithm 1:

**Algorithm 1** Data acquisition by modifying the image nature. (Figure 6 top). **Input:** Capture image **Output:** Extract CoG(X,Y),β values**Require:** camera ON1:Capture (Img)2:Binarize (Img) //*Threshold of the object to be detected*3:**if** (Object_detected (Img) == **true**) **then**4: //*Image Pre-Processing*5: Set_lines = Hough_lines(Img)6: θ = Pers_Angle_Estimation(Set_lines) //*Calculation of perspective angle*7: Img_rect = Rot_pers(img,θ) //*Image rotation*8: 9: //*Parameter computation*10: Bottle = Get_Blobs(Img_rect)11: $\left(X_{CoG},Y_{CoG}\right)$ = Get_CoG(Bottle)12: β = θ13: **end if**14: **return** $(X_{CoG},Y_{CoG}),\beta$



As it can be seen in this algorithm, to obtain the required values of the bottle by this procedure, the image is captured from the camera (Binarize(Img)). Then, a filter is applied to get the object of interest (in this case, the bottle) (Object_detected(Img)). In the next step, the Hough lines transformation is used (Set_lines=Hough_lines(Img)) to obtain the lines of the bounding box of the bottle. This operation implies a time complexity of $O{\left(N\right)}^{3}$). Then, the angle of the lines is computed (θ=Pers_Angle_Estimation(Set_lines)) and the image is rotated this angle (Img_rect=Rot_pers(img,θ)). This operation is not only applied to the object of interest but to the whole image, masking other useful information. In the last part of this algorithm (parameter computation), the bottle area is extracted (Bottle=Get_Blobs(Img_rect)) to compute the CoG ($\left(X_{CoG},Y_{CoG}\right)=Get\_CoG\left(Bottle\right)$). Finally, the inclination angle is equal to the angle obtained with the Hough transformation. (β=θ). Using this transformation, the perspective errors in the image cannot be corrected. Because of this, the angle obtained has big distortion. In the following sections of this paper, another way to obtain the information is going to be presented in order to have a lower time complexity to obtain control of the bottle and lesser errors.

### 3.2. Linear Inverted Pendulum Model (LIPM)

To achieve the equilibrium control and to know which information is needed for that purpose, it was mandatory to model the behavior of the bottle or drinks on the tray. The model chosen to represent the bottle performance is an inverted pendulum. Based on this model, the robot knows the right way to use its own arm for balance control. Any kind of perturbation, over the bottle or over the robot, causes an overturning moment that will be detected by the robot’s wrist sensors. The relative position between the robot and the bottle is only important from the waiter task kinematics’ point of view. That is, the proposed task consists of maintaining the tray in a horizontal position independently of the robot’s posture. Once the external perturbation is applied on the system, the robot corrects the instability of the object, maintaining its balance on the tray.

The Linear Inverted Pendulum Model (LIPM) [15] has been chosen as the model to define the behavior of the system, due to the similitude. In both cases, that of the bottle and the model, the Center of Mass (CoM) is above its pivot point. The forces described by this model are shown in Figure 7 and Equation (1):

(1) $$\tau =ml^{2}\ddot{\theta }-mgl\theta ,$$

where $Z_{C}$ is the constant height characteristic of the LIPM. τ is the torque applied on the joint to control the balance. $\ddot{\theta }$ and θ are the angular acceleration and position of the pendulum. *m* and *l* are the mass and the length of the CoM of the pendulum. Finally, *g* is the gravity. Considering only visual measurements, it has been assumed that the CoG of the bottle is close to the CoM. Then, both points are coincident ($X_{CoG,B}\equiv X_{CoM,B}$).

In the case described above (Figure 8), the bottle does not rest on a single point, for example, the bottle rests on a surface. Therefore, it is necessary to apply an strategy to define the state of stability of the bottle using the ZMP as an indicative. Thus, when the projection of the sum of the forces/torque in the CoM of the bottle exceeds the support surface with the tray [6], it will fall. In order to calculate the projection of ZMP, the values of position and inclination of the bottle generated with the camera are used once they have been corrected by the methods discussed in this paper. Of course, all of these values are constantly being calculated through artificial vision, while the arm tries to maintain the stability of the bottle (Figure 8). This implied that the algorithm’s time complexity is an important factor to take into account to achieve a good control.

In previous research, like the experiments carried out in [16], the estimated future positions obtained by vision are obtained. In the case presented in this paper, the maximum angle has been calculated by the use of trigonometric equations. The bottle has been placed over a non-slippery surface. Therefore, as it can be seen in Figure 9, the bottle loses equilibrium once the CoG overtakes the limit of the rest surface.

In Figure 9, the blue object is placed on a plane surface. The red figure represents the bottle positioned at the equilibrium limit. The CoM is moved the equivalent to the β angle. The length and width data (L and W, respectively) of the bottle have to be used to obtain this angle. As the CoG is in the center, if the tangent is calculated, the alpha angle can be calculated using Equation (2):

(2) $$\alpha =\arctan\frac{L}{W}=71.995\deg≃72\deg,$$

where *L* corresponds to the length of the bottle and *W* is the width of the bottle. Once the α angle has been calculated, the β angle is obtained by subtracting alpha from 90°, where β is the complementary angle to α, and it is also the limit tilt angle:

(3) β=90deg−α=108deg.

As shown in Equation (3), the bottle loses the equilibrium once it overtakes the angle 72° and 108°. To compensate for possible variations in the CoM and dynamic-reactions of the system (Equation (1)), a safety coefficient has been applied to that angle. Those new conservative limits are set as 80° and 100°. The angle space range is configured in such a way that zero degrees represents the bottle lying completely to the right, 90 degrees represents the bottle being totally straight and 180 degrees represents when it is lying on its left side. Considering that range, the acceptable error that has been configured is 0.5 degrees. This error has been chosen because a derivation of 0.5 degrees is not critical for the bottle equilibrium, considering a spectrum of 180 degrees.

This pendulum model without the car has been used due to large friction forces between the bottle and the tray. Due to the configuration of the tray without non-slip material, it can be affirmed that there will be no linear movement between the bottle and the tray. Only rotational movements will be generated. For this reason, the use of the LIPM was chosen instead of the cart-table model. Therefore, the control algorithm mainly focuses on variations of the rotation’s angle of the bottle to indicate its stability. In addition, in the mathematical pendulum model, the viscosity can be disregarded. This allows us to simplify the model further and, thus, to reduce the computing load during the control of the bottle.

### 3.3. Neuro-Fuzzy Learning

The use of Fuzzy systems presents multiple advantages, being a choice for much control research in soft computing. Some of these advantages are the use of real information of the system to be controlled, its good resistance to the noise, or the low computation effort. For example, in the research carried in [17], the Fuzzy is used to control a manipulation system. Another example of the Fuzzy usage is [18], in which the high amount of information is processed by using the Fuzzy C-means as a clustering algorithm. In addition, the capability of the Fuzzy filters to come up against nonlinear systems is useful. Therefore, it is perfect to build motor controllers and fight against nonlinearities caused by the motor system or its load [19]. The application range of the Fuzzy is so wide that it is not only being applied on control systems, but it also has been used in schedulers to find the paths in grids with higher accuracy and convergence velocity [20].

To train the Fuzzy filter used in the error correction, Neural Learning has been implemented. Therefore, it is important to explain how the Neuro-Fuzzy Learning works. The adaptive Neuro-Fuzzy Inference System (ANFIS) [21] is a multilayer feed-forward network in which each node performs a particular function on incoming signals as well as a set of parameters belonging to this node. The equations of the node functions may vary from node to node, and the choice of each node function depends on the overall input–output function that the adaptive network is required to perform. To reflect different adaptive capabilities in an ANFIS network architecture, both circle and square nodes are used. A square node (adaptive node) has parameters while a circle node (fixed node) has none [22,23], and this graphic representation can be seen in Figure 10 inside the block of “Neuro Training”.

Suppose that a given adaptive network has *L* layers and the $k^{th}$ layer has r(k) nodes. The node can be denoted in the $i^{th}$ position of the $k^{th}$ layer by (k,i), and its node function (or node output) by $O_{i}^{k}$. Since a node output depends on its incoming signals and its parameter set, then $O_{i}^{k}$ can be preseted with Equation (4):

(4) $$O_{i}^{k}=O_{i}^{k}\left(O_{i}^{k-1},\ldots ,O_{r(k-1)}^{k-1},a,b,c,\ldots \right),$$

where a,b,c, etc., are the parameters belonging to this node. Assuming that the given training data set has *P* entries, the error measure can be defined for the $p^{th}$ (1≤p≤P) entry of training data entry as the sum of squared errors, as can be shown in Equation (5):

(5) $$E_{p}=\sum_{m=1}^{r(L)}{\left(T_{m,p}-O_{m,p}^{L}\right)}^{2},$$

where $T_{m,p}$ is the $m^{th}$ component of $k^{th}$ target output vector, and $O_{m,p}^{L}$ is the $m^{th}$ component of actual output vector produced by the presentation of the $p^{th}$ input vector. Accordingly, the update formula (Equation (6)) of the training parameters, the generic parameter λ (parameter of the given adaptive network) is:

(6) $$△\lambda =-\eta \frac{\partial E}{\partial \lambda },$$

in which η is a learning rate that can be further expressed as Equation (7):

(7) $$\eta =\frac{k}{\sqrt{\sum_{\lambda }{\left(\frac{\partial E}{\partial \lambda }\right)}^{2}}}.$$

Hence, the overall error measure is:

(8) $$E=\sum_{p=1}^{P}E_{p}.$$

The training process ends when the overall error *E* is less than or equal to a pre-established threshold value ξ at the $n^{th}$ iteration. Otherwise, the neuro training should be performed again, maybe with a higher amount of samples, by choosing ones more representatives or with more accurate information. The result is the set of functions of the output node $O_{i}$:

(9) $$if\;\;{\left(E\leq \xi \right)}_{n}⟶{\left(O_{i}\right)}_{n}.$$

When the training process has finished the Fuzzy filter is ready, as shown in Figure 10 (bottom). The Fuzzy filter consists of a set of if-then rules, in the form of Equation (10). The input parameters $p_{n}$ are compared with the corresponding bounding values ($A_{n}$,$B_{n}$) that have been established for each parameter. The output of the Fuzzy filter is one value ($\hat{C}$) that accomplishes with all conditions inferred by the rules.

(10) $$if\;\;\left(A_{1}<p_{1}<B_{1}\right)\;\;and\;\cdots \;\;\left(A_{n}<p_{n}<B_{n}\right),\;\;then\;\;\hat{C}.$$

The output value of the Fuzzy approximation must be less than the training threshold (Equation (11)). This should be the maximum error committed by the Fuzzy filter for each input set:

(11) $$|C-\hat{C}|\leq \xi .$$

## 4. Methods and Experimental Procedure

To obtain the information needed to make the corrections in the image and to remove the errors caused by the perspective deformation and the ones caused by the camera lenses, an experimental set-up has been defined. In this section, we explain the steps followed in this set-up and the way the information for the Neuro-Fuzzy filter has been obtained.

As it was said in the background and the problem statement sections, in other existing techniques, irregardless of the real inclination or orientation, the operations used to correct the perspective error in the images modify the object nature in such a way that the bottle finally appears fully straight. This variation in the obtained information, as shown in the top section of Figure 6 named Classical Approaches for Object Detection, leads into a lack of knowledge of the real inclination angle of the bottle, making it unsuitable for our application. As a consequence, it is not possible to achieve arm control to maintain the bottle in equilibrium.

If it is this way, in order to obtain the real angle of the bottle, several trigonometric operations must be added to the operations previously performed over the bottle image. This would lead to an increase in the computational cost due to the high time complexity of the algorithms [24]. As it was explained in the subsection of vision inside the background, the operations that are performed to modify the image have a computational cost with an order of $O{\left(n\right)}^{3}$. This makes these procedures infeasible for our proposes.

Therefore, instead of making a correction on the image based on trigonometric calculus, which takes too much time, an alternative to these classical methods is proposed in this paper, avoiding the complex image manipulation steps needed in the other procedures.

As seen in Figure 6 in the green section called New Approach Proposed, on the proposed approach, the image is not corrected, maintaining the wrong information in the image. However, we correct the perspective errors and the camera errors that have a previous knowledge of the real angle of the bottle. These reference data are recovered in datasets, by performing several experiments that are used later in a Neuro-Fuzzy learning system to model the error. Once the Neuro-Fuzzy filter has been trained, the error introduced in each point of the image acquired by the camera is known.

The diagram shown in Figure 11 represents the procedure followed to develop the ANFIS. First of all, the information needed to train the Neuro-Fuzzy filter has been obtained. To acquire the data, several sweeps have been done, positioning the bottle in front of the robot camera and obtaining “n-IMAGES”. These sweeps were done by configuring the bottle in controlled and known inclination angles. From the images obtained, the characteristics needed to close the control loop have been obtained “m-FEATURE” (position of the geometrical centre of the bottle (x,y), inclination angle of the bottle). As is known, those angle features are mixed with the camera errors.

However, as the data was recovered, knowing the real angle of the bottle, it is possible to have knowledge of the error, which is introduced in each position of the image. With all of this information, both a “TRAINING DATASET” and a “CHECKING DATASET” have been created.

On the one hand, in the training dataset, the behaviour of the error introduced in the inclination angle among the image space has been recovered in a table. With this data, the error has been modelled, and it contains information to correct it later. In contrast, in the second dataset, the information has been obtained from other different positions of the space in the image, adding knowledge about the whole vision field. In those datasets, the real inclination angles are well known, having a relation between the real angles and the angles obtained by vision. This information allows us to check if the ANFIS is reliable.

To develop a robust system, it it necessary to train it with different configurations. A total of 50 samplings in a wide range of positions have been made, acquiring more than 20,000 data points that have been introduced in the training and checking datasets. The number of iterations has been changed, starting with a low number and increasing it in the next tests in order to find the right number of iterations to train the system properly.

Once the Neuro-Fuzzy filter has been trained with the information of the error regarding each of the positions set in the dataset, it is capable of doing a correction in the new information obtained with the camera. In this step, the program implemented in TEO is the Fuzzy filter obtained, the behaviour of which corresponds to the one shown in the Algorithm 2:

**Algorithm 2** Data acquisition by using a Neuro-Fuzzy filter. (Figure 6 bottom). **Input:** capture image **Output:** extract ${\hat{\alpha }}_{real},CoG\left(X,Y\right)$ values**Require:** camera ON
1:Capture (Img)2:Binarize (Img) //*Threshold of the object to be detected*3:**if** (Object_detected (Img) == **true**) **then**4: //*Extraction values from the original frame*5: Bottle = Get_Blobs(Img)6: ($X_{CoG},Y_{CoG}$) = Get_CoG(Bottle)7: α = Get_Angle_Inclination(Bottle)8:**end if**9: 10://*Applying of the Fuzzy filter previously trained*11:**while** (*i* < *n*) **do**12: //*Search of similarities within the dataset*13: **if** (($Li_{n}<X_{CoG}<Ls_{n}$) **and** ($Ri_{n}<Y_{CoG}<Rs_{n}$) **and** ($Pi_{n}<\alpha <Ps_{n}$)) **then**14:    ${\hat{\alpha }}_{real}=Angle_{error}-\alpha$15:    **return**16: **end if**17: *i*++18:**end while**19:**return** $X_{CoG},Y_{CoG},{\hat{\alpha }}_{real}$ // *${\hat{\alpha }}_{real}$ is the real inclination angle*


As it can be deduced from the pseudo-code presented before, the image captured by the camera (Capture(Img)) is pre-processed (Binarize(Img)) to isolate the bottle (Object_detected(Img)). With this image, the bottle area is computed (Bottle=Get_Blobs(Img)), the CoG position is obtained ($X_{CoG},Y_{CoG}=Get\_CoG\left(Bottle\right)$), and the inclination angle (α=Get_Angle_Inclination(Bottle)) are extracted. However, this angle still has the intrinsic errors of the process of capturing the image. Thus, the trained Fuzzy filter is applied. The location of the bottle (CoG) and the inclination detected are the inputs to the Fuzzy filter. This process allocates an error (ϵ) according to bottle location, as depicted in Figure 12. The output from the filter is the error of the angle ($Angle_{error}$) that must be subtracted from the previous inclination angle (α) to obtain the real one (${\hat{\alpha }}_{real}$). In this case, the Fuzzy filter time complexity corresponds to linear time O(n) [25], as far as it depends on the amount of data (*n*) that was previously included in the training dataset. The time complexity is lower than that of the “Classical approaches”.

During the data acquisition, we have established a priority in the information obtained. As seen in Figure 12, the images have been divided into nine zones. This division will help to achieve a robust human inspired control [26] of the equilibrium of a bottle on a tray in the future. The main reason behind this division is: after having evaluated all the acquired information, a relationship has been shown. The further the bottle is from the center of the image (Zone B2), the higher the distortion error is. This relation also appears when the results are evaluated in the three different rows; the central one has the lower distortion. As a consequence, the TEO’s head has been configured to move in such a way that the bottle is maintained most of the time in the center of the image (Zone B2) or in the three central zones of the image (A2, B2, C2). Thus, it can be said that these nine different zones have been defined according to the accuracy in the correction required for each one of them.

The quadrant division has been defined considering the main positions that the bottle occupies in the image. Because the head of the robot has been programmed in such a way that, when it moves, it tries to keep the bottle centered in the divisions A2, B2 and C2 seen in Figure 12, and the bottle is positioned most of the time in the three horizontal central quadrants. As those quadrants are occupied by the bottle most of the time, the accuracy in the error correction is higher inside them. As the central quadrant has the highest rate of cases in which the bottle is positioned, the error there must be the lowest in the image.

Following these statements, it is understandable that, to obtain the samplings needed for the training dataset, the amount of information obtained is higher in the red and yellow zones rather than in the green ones. The goal is to reduce the error in those positions as much as possible. Therefore, in those positions, several sweeps with a wide range of inclination angles of the bottle have been made, whereas in the green ones a lower amount of data was needed for the training dataset.

All of this information has been introduced in the Adaptive Neuro-Fuzzy Inference System (ANFIS), available in $Matlab^{TM}$ *R2017b* (The MathWorks, Inc., USA) With the ANFIS, the Fuzzy filter was obtained. The filter is used to make the corrections in the inclination angle of the bottle perceived by the camera. With this correction, the real tilt angle information needed to make the proper rectification of the tray position is finally achieved.

## 5. Experiments and Results

In this section, we will describe the experiments that have been carried out to prove the validity of the Fuzzy filter developed. The common procedure followed during the experiments has consisted of positioning the bottles in the different areas depicted in Figure 12. Then, the image of the bottle has been captured and the algorithm proposed has been applied to obtain the bottle angle. Two different experiments have been performed.

### 5.1. First Experiment: Constant Bottle Angle in the Main Visual Area

In this first experiment, several sweeps in the central horizontal row were done as shown in Figure 13. During these sweeps, the bottle was maintained fully straight, with an angle of 90 degrees in relation to the horizontal plane, keeping the bottle straight and keeping the axis of the pixel coordinate Y constant.

The images obtained with the robot camera were post-processed following the steps named and the information extracted from those images was stored in an input dataset, which was then processed by the Fuzzy filter. Again, this dataset is made up by the position of the geometrical center of the bottle in the pixel X and pixel Y, and the inclination angle perceived by the camera. After having processed this information through the filter, the estimated real tilt angle of the bottle has been obtained. In the graphic shown in Figure 14, the *y*-axis represents the inclination angles, whereas the *x*-axis represents the different existing pixels in the *x*-axis of the image. In this graphic, two lines can be seen. The blue one represents the angle perceived by the humanoid robot in the different positions of the pixel coordinate X. It can be seen that, when the bottle is next to the lateral limits of the image, the error that is introduced increases. The real angle of the bottle in the experiment was kept constant (90 degrees). However, while the bottle is next to the center of the image, this error disappears and some correction is needed. Due to the explanation related to Figure 12, the behavior of the information acts as expected. Considering that the obtained images have about 283 pixels in the *x*-axis, if we divide it into three quadrants, the amount of pixels per quadrant would be about 95. Therefore, it can be seen in the graphic that the error is higher in the lateral ones. In addition, this blue signal is also very noisy. The origin of this instability is caused by the error introduced in the vision systems by the illumination conditions, which can affect the recognition of the bottle.

After having post-processed the information with the Fuzzy filter, the error was corrected and the 90 degrees angle has been obtained. This corrected information is shown by the red line. As it can be seen in the graph, this red line is really close to 90 degrees. Knowing that the experiment has been performed with a real angle of the bottle equal to 90 degrees, we evaluate the higher value of the red line and calculate that the error of the system in this experiment is not higher than 0.0172 degrees. This error completely fulfills our established requirement of obtaining an angle with an error below 0.5 degrees.

### 5.2. Second Experiment: Positioning the Bottle with the Robot Arm

Once the Fuzzy filter has been proven to be reliable in a controlled situation, such as an horizontal sweep, a second and more complex experiment has been performed. In this case, the bottle has been placed on the tray grasped by the robot and the robot has moved the arm in such a way that the bottle has been placed in the different quadrants in which the image has been divided.

This second experiment has been carried out by positioning the bottle in different inclinations, which were also controlled and known. The goal pursued in this second experiment is to test if the Fuzzy filter is also capable of properly rectifying the data obtained by the robot camera, despite the inclination angle of the bottle and the quadrant where the bottle is in that moment.

In this case, the bottle has been placed on the tray that the robot is holding and the robot arm has moved it along the room (Figure 15). All of those movements of the bottle have been recorded by the robot camera, which is an *ASUS Xtion PRO* $LIVE^{TM}$ camera (ASUSTeK Computer Inc., Taiwan). From these movements, the information related to the position in the pixel coordinate X (column PIXEL X) and the pixel coordinate Y (PIXEL Y), as well as the inclination angle acquired by the camera (ANGLE DETECTED), are obtained and recorded in Table 1. It is important to remark that the tilt angle obtained (ANGLE DETECTED) is not the real one (REAL ANGLE).

The information stored in this table has been processed by the Fuzzy filter, which applies the corresponding correction in the inclination angle detected for the bottle using the filter created by the Neuro-Fuzzy ANFIS. After applying the correction corresponding to the error surface (FUZZY CORRECTION), new data are recovered in a new column in Table 2 (CORRECTED ANGLE). This processed information corresponds to the real inclination angle of the bottle at each moment.

To prove that the work of this Fuzzy filter is correct, the bottle has been positioned in known inclinations, in such a way that the data obtained after processing the image can be compared with the real one. The result gives real information of the error introduced, taking into account the procedure proposed in this paper.

As can be seen in the results shown in Table 3, the angle obtained firstly by the camera differs from the real one. However, once the information has been processed, the results of the inclination angle of the bottle are quite similar to the real tilt angles of the bottle. It is true that, in some cases, there is still a percentage of error. However, if we compare the error of the information obtained directly from the camera (% CAMERA ERR) with the error of the angle after having applied the fuzzy filter (% FUZZY ERR), it can be seen that the error has been drastically decreased.

In a representative way, in the three tables, nine experiments have been shown above. There, the relation between the data obtained just by processing the camera image and the data after applying the correction from the Fuzzy filter can be seen. The points shown in the table have been obtained with different inclinations of the bottle and changing the positions of the bottle in the image, in such a way that there are nine representative samples of the different situations that can be found in a real experiment.

## 6. Conclusions

In this work, it has been stated the need of improving control algorithms, especially in balance’s control due to its fast dynamics. The increase of the velocity of data processing and the quality of the resulting information is one way of achieving a proper control. The task presented, in which the humanoid robot transports a bottle on a tray, is a clear example of a balance control task. This task needs accurate sensor information and it needs to obtain the information as fast as possible.

The results presented show the validity of the experimental procedure to develop a Fuzzy filter to gather all the visual computing information in one step. On the one hand, it has been shown that the method is faster than other classical approaches implying a lower computational cost. On the other hand, in the experiments carried out, the error obtained by the camera has been corrected or reduced drastically in comparison to the original error. Despite this, there is still an existing error in the data. However, it is true that this error can further be reduced by increasing the data obtained to improve the training of the Neuro-Fuzzy system. It can be concluded that the results obtained are satisfactory enough for the intended purposes. The proposed method is capable of obtaining the estimated inclination angle with a low percentage of error. For that reason, proving that the method explained is adequate to correct visual perception errors was an important milestone. However, the work discussed has been oriented to obtain the error pattern shown in Figure 12. In the future, this pattern can be modified depending on the application in order to get another pattern that could provide a better answer to the system.

As stated before, this research has been developed as a part of the future goal of achieving a waiter robot that is able to control the equilibrium of a bottle on a tray. The improvement introduced with the development of the Fuzzy filter allows the modification of the control architecture, making it more simple. It allows for facing other computing problems more related to low-level control than sensor parameters’ extraction. In addition, the experimental procedure followed is applicable to other sensory sources and it allows for mixing data from a different nature. For example, in the application described, visual and Force-Torque data of the bottle will be used for controlling balance. These totally different data sources can be combined to enrich the information provided by the output of the Fuzzy filter. The result would be a complex parameter that, combined with the simple models used for balance control, would improve the controller performance.

The use of this methodology could also be extended for robot’s pose control. In this way, based on visual methods, it would be possible to estimate the robot’s arm position and orientation without relying on complex inverse kinematics computation. If enough accuracy is obtained, the use of a Fuzzy filter for kinematics would allow the development of new control algorithms for fast response.

## Figures and Tables

**Figure 1:**
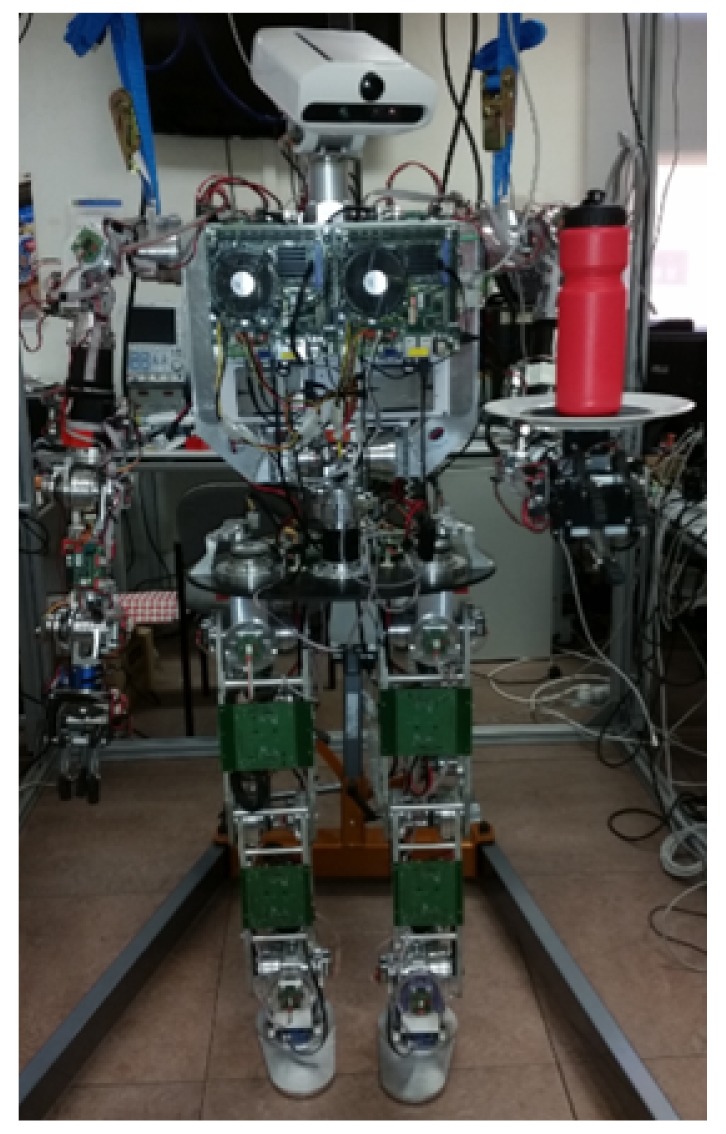
Humanoid robot TEO (Task Environment Operator) transporting an object.

**Figure 2:**
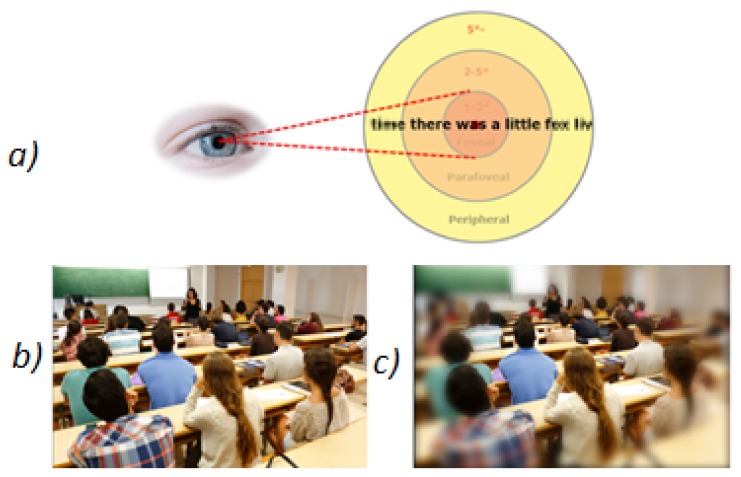
Comparison between an image obtained by computer vision vs. human vision.

**Figure 3:**
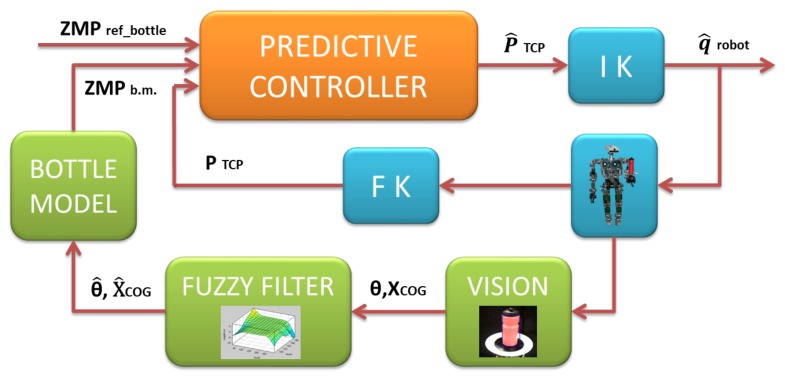
Control of the equilibrium of a bottle on a tray in a Non-Grasping task.

**Figure 4:**
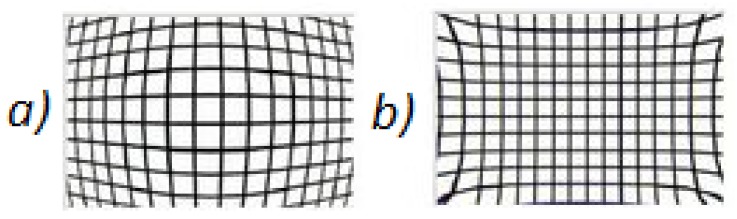
Image deformation caused by the camera lenses.

**Figure 5:**
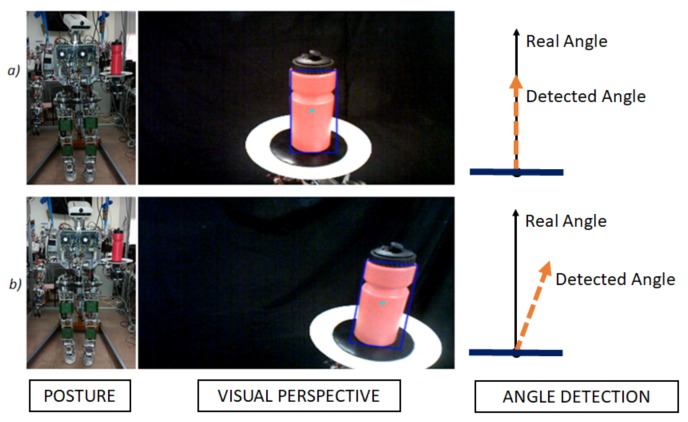
Perspective error, the same tilt angle changing the camera position.

**Figure 6:**
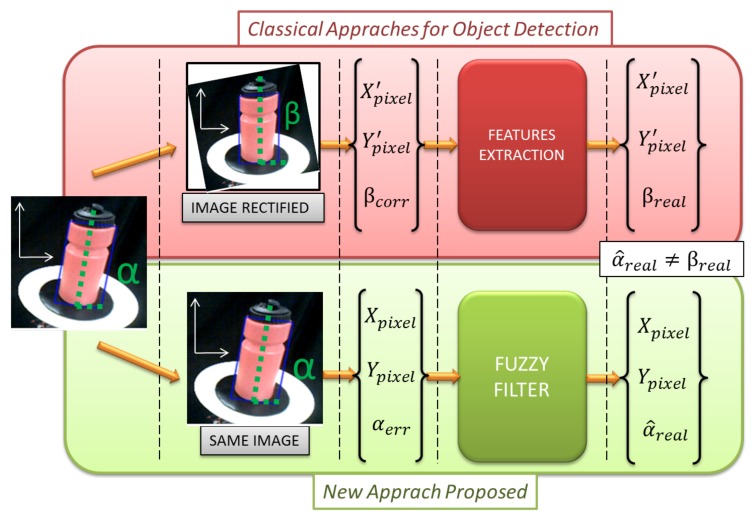
Comparison between classical approaches and the proposed approach in this paper.

**Figure 7:**
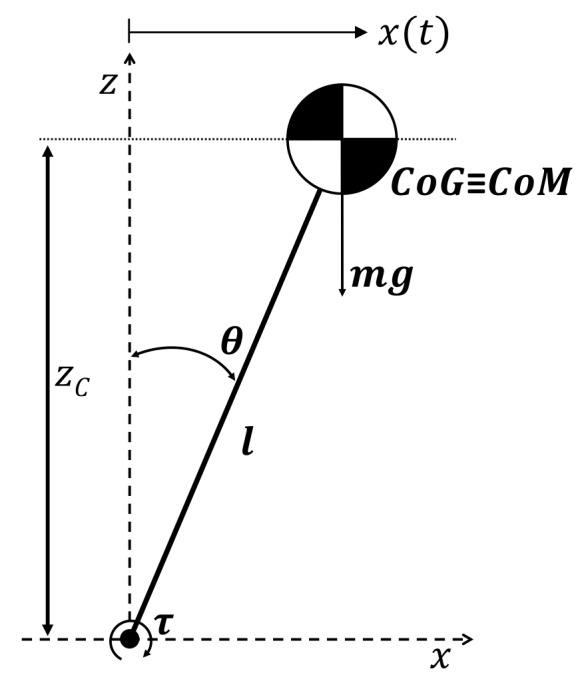
Linear Inverted Pendulum Model (LIPM).

**Figure 8:**
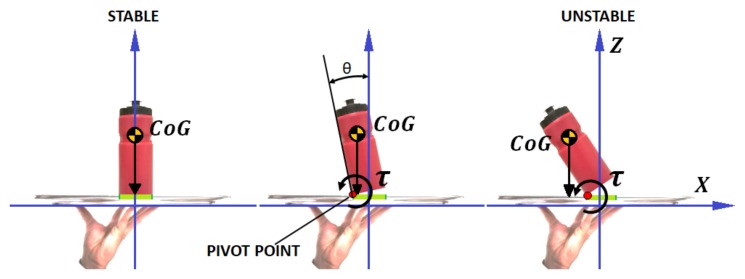
Different degrees of stability applied to the LIPM model.

**Figure 9:**
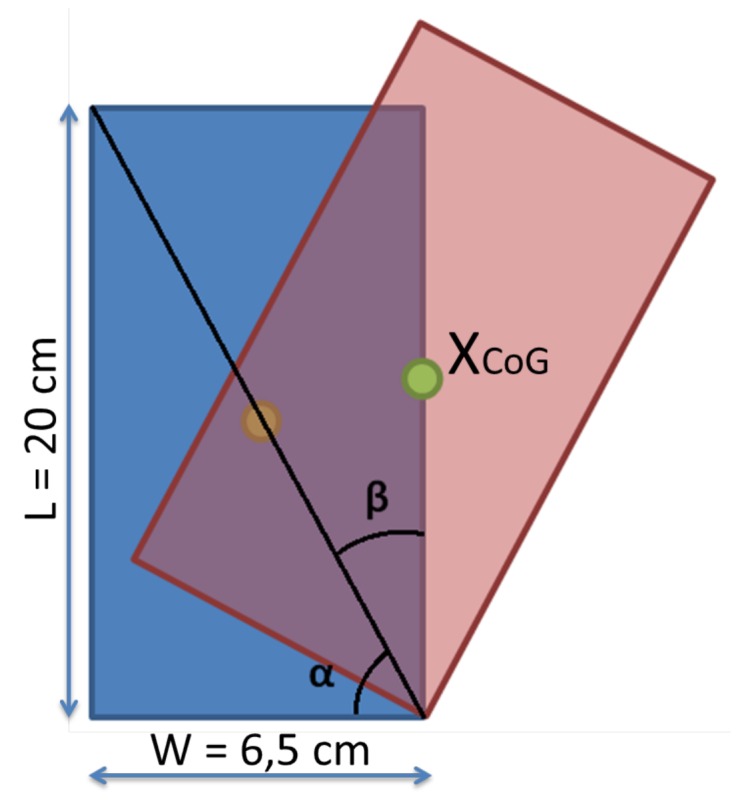
Scheme representing the angles that must be obtained to calculate the limit tilt angle in which the bottle can be placed before falling.

**Figure 10:**
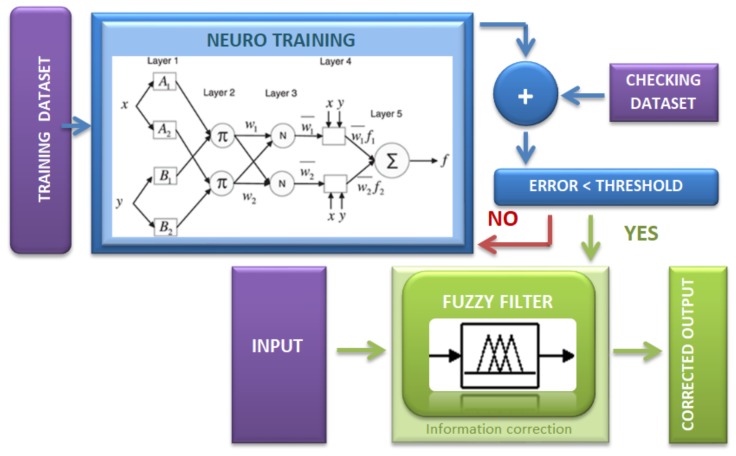
Adaptive Neuro-Fuzzy Inference System (ANFIS) network architecture.

**Figure 11:**
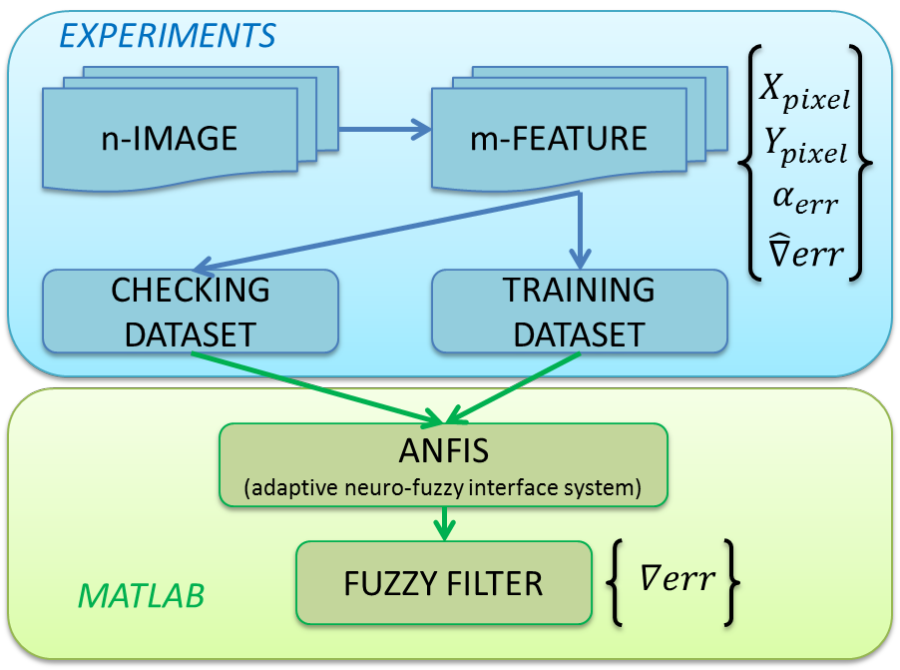
Diagram corresponding to the experimental set-up followed in the experiments performed.

**Figure 12:**
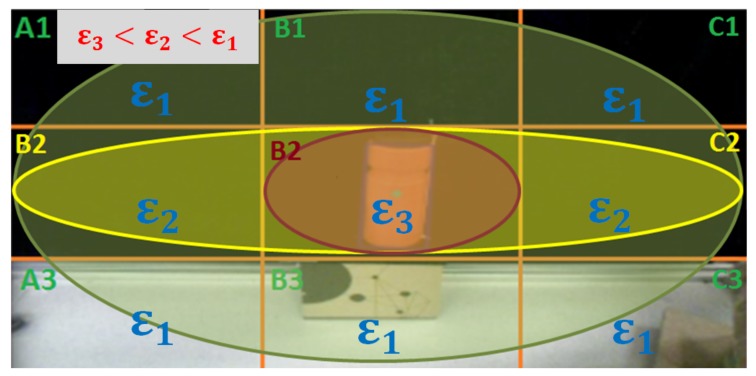
Selection of the Error Priority in the different quadrants of the Image.

**Figure 13:**
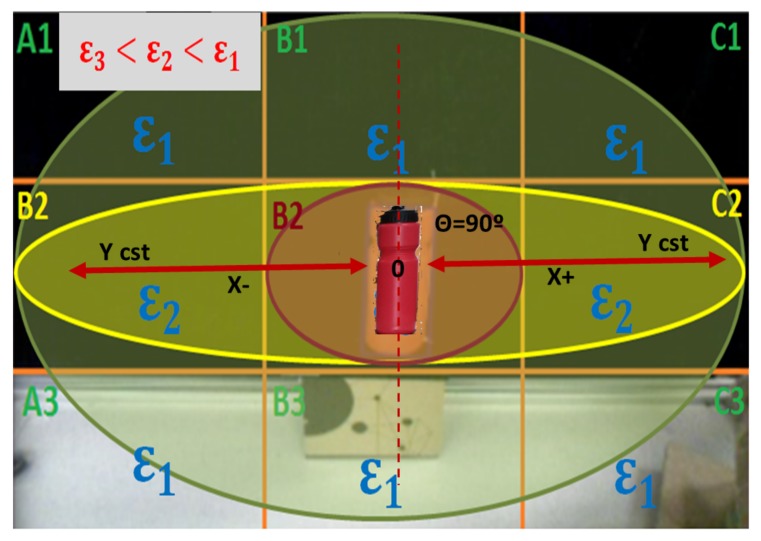
First experiment.

**Figure 14:**
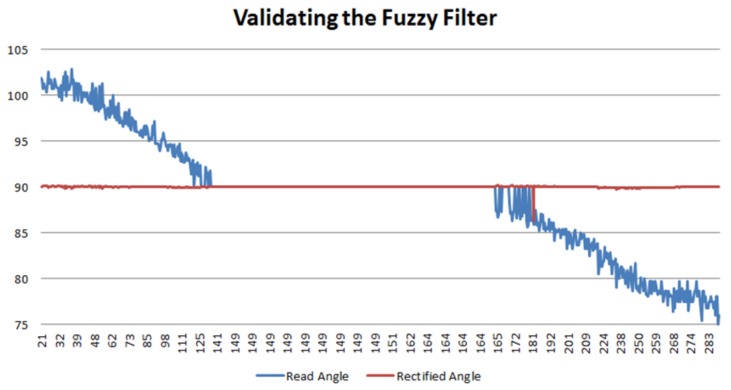
Results from the first experiment.

**Figure 15:**
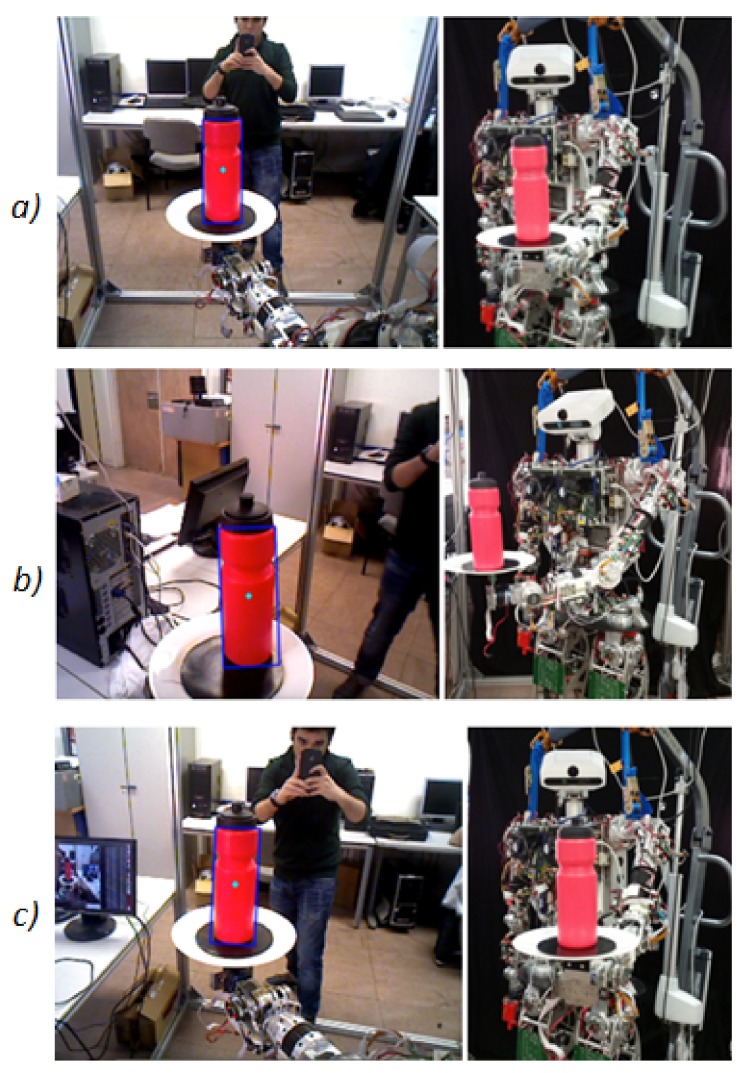
Second experiment. Positioning the bottle in different inclination angles and positions. (**a**) Bottle at robot left side (inclination 87 degrees); (**b**) Bottle at the front of the robot (inclination 90 degrees); (**c**) Bottle at robot left side (inclination 90degrees).

**Table 1:** Results obtained after having processed the information through the Fuzzy filter.

POINT	PIXEL X	PIXEL Y	REAL ANGLE	ANGLE DETECTED
P1	270	157	81	68.159
P2	26	62	81	93.0146
P3	294	149	99	83.405
P4	44	66	99	107.354
P5	282	152	87	74.0545
P6	43	69	87	98.7461
P7	286	148	93	76.7594
P8	35	62	93	103.3924
P9	131	67	90	91.1691

**Table 2:** Results obtained after having processed the information through the Fuzzy filter.

POINT	FUZZY CORRECTION	CORRECTED ANGLE
P1	−18.5025	86.6615
P2	8.6881	84.3265
P3	−6.3794	89.7844
P4	20.4483	86.9057
P5	−13.3798	87.4343
P6	9.0118	89.7343
P7	−13.2984	90.0578
P8	12.816	90.5764
P9	0.9414	90.2277

**Table 3:** Results obtained after having processed the information through the Fuzzy filter.

POINT	R. ANGLE	ANGLE DETECTED	% CAMERA ERR	CORRECTED ANGLE	% FUZZY ERR
P1	81	68.159	15.83	86.6615	6.9
P2	81	93.0146	14.83	84.3265	4.1
P3	99	83.405	15.75	89.7844	9.3
P4	99	107.354	8.43	86.9057	12.2
P5	87	74.0545	14.87	87.4343	0.5
P6	87	98.7461	13.50	89.7343	3.1
P7	93	76.7594	17.46	90.0578	3.2
P8	93	103.3924	11.17	90.5764	2.6
P9	90	91.1691	1.29	90.2277	0.2

## Abbreviations

The following abbreviations are used in this manuscript:

TEO	Task Environment Operator
LIPM	Linear Inverted Pendulum Model
ZMP	Zero Moment Point
CoM	Center Of Mass
CoG	Center of Geometry
ANFIS	Adaptative Neuro-Fuzzy Inference System
